# Fullerene derivatives with oligoethylene–glycol side chains: an investigation on the origin of their outstanding transport properties†

**DOI:** 10.1039/d1tc02753k

**Published:** 2021-08-18

**Authors:** Jingjin Dong, Selim Sami, Daniel M. Balazs, Riccardo Alessandri, Fatimeh Jahani, Li Qiu, Siewert J. Marrink, Remco W. A. Havenith, Jan C. Hummelen, Maria A. Loi, Giuseppe Portale

**Affiliations:** Zernike Institute for Advanced Materials, University of Groningen, Nijenborgh 4 Groningen 9747AG The Netherlands g.portale@rug.nl; Stratingh Institute for Chemistry, University of Groningen, Nijenborgh 4 Groningen 9747AG The Netherlands; Groningen Biomolecular Sciences and Biotechnology Institute, University of Groningen, Nijenborgh 7 Groningen 9747AG The Netherlands; Department of Chemistry, Ghent University, Krijgslaan 281 (S3) Gent B-9000 Belgium

## Abstract

For many years, fullerene derivatives have been the main n-type material of organic electronics and optoelectronics. Recently, fullerene derivatives functionalized with ethylene glycol (EG) side chains have been showing important properties such as enhanced dielectric constants, facile doping and enhanced self-assembly capabilities. Here, we have prepared field-effect transistors using a series of these fullerene derivatives equipped with EG side chains of different lengths. Transport data show the beneficial effect of increasing the EG side chain. In order to understand the material properties, full structural determination of these fullerene derivatives has been achieved by coupling the X-ray data with molecular dynamics (MD) simulations. The increase in transport properties is paired with the formation of extended layered structures, efficient molecular packing and an increase in the crystallite alignment. The layer-like structure is composed of conducting layers, containing of closely packed C_60_ balls approaching the inter-distance of 1 nm, that are separated by well-defined EG layers, where the EG chains are rather splayed with the chain direction almost perpendicular to the layer normal. Such a layered structure appears highly ordered and highly aligned with the C_60_ planes oriented parallel to the substrate in the thin film configuration. The order inside the thin film increases with the EG chain length, allowing the systems to achieve mobilities as high as 0.053 cm^2^ V^−1^ s^−1^. Our work elucidates the structure of these interesting semiconducting organic molecules and shows that the synergistic use of X-ray structural analysis and MD simulations is a powerful tool to identify the structure of thin organic films for optoelectronic applications.

## Abstract 2

The synergistic use of X-ray scattering and molecular dynamics simulations reveals the structure–property relationships of [60]fullerene derivatives with oligoethylene–glycol side chains.

## Graphical abstract

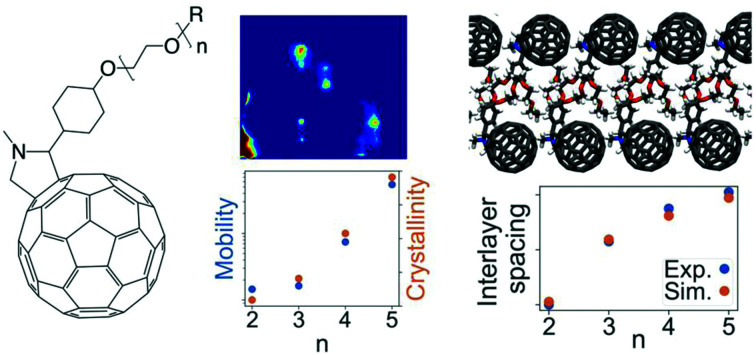

## Introduction

For a long time, [60]fullerene (C_60_) and its derivatives have been at the core of the development of the organic electronics field. This is the field that for a couple of decades has advocated the use of organic molecules and polymers, mostly processed from solution, for the fabrication of a new generation of electronic and optoelectronic devices, characterized by mechanical flexibility, lightweight properties and cheap fabrication technology. In this community, these carbon allotropes have been praised not only because of their fascinating structural and physical properties but also because, initially, they were one of the few molecular systems that could display substantial n-type transport due to their strong electron affinity. However, in its pristine form, the C_60_ molecule has very low solubility and cannot give the flexibility of use that was initially envisioned. The development of fullerene chemistry,^1^ and the multitude of solution-processable derivatives that were synthesized using those methods were the definitive boost for their use and for the enthusiasm of a large community of scientists and engineers for these molecules. At that point, fullerenes became a common building block of many types of devices, one of the most popular being the phenyl–C_61_–butyric acid methyl ester (PCBM) derivative,^2^ not only together with other organic compounds like in organic solar cells,^3,4^ but also used on top of silicon and GaAs.^5–7^ Nowadays, while being still very popular in organic solar cells together with the new generation of organic acceptors called non-fullerene acceptors,^8^ fullerenes have also found a large space in metal halide perovskite solar cells as electron-transport layers and passivating agents.^9^ Furthermore, they are key in organic thermoelectrics^10,11^ and have been investigated for medical applications.^12,13^

When looking at the transport properties of fullerene derivatives, it is rather obvious that the side chains and the functional groups are not simply innocent spectators. It has been widely reported and accepted that many fullerene derivative thin films have layered structures with high crystallinity and orientation.^14,15^ On this basis, side branches are found to play a key role in the film structure and thus have an impact on the transport properties. In 2005, Chikamatsu *et al.* studied the long-chain alkyl-substituted C_60_ derivative, C_60_-fused *N*-methylpyrrolidine-*meta*-C12 phenyl (C_60_MC12), and found that the self-assembling ability of the side chains is crucial for the formation of highly ordered crystalline films.^16^ Later, the same research group reported that the side chain orientation is also very important.^17^ By comparing C_60_-fused *N*-methylpyrrolidine-*ortho*-C12 phenyl (C_60_OC12), C_60_MC12, C_60_-fused *N*-methylpyrrolidine-*para*-C12 phenyl (C_60_PC12), and C_60_-fused *N*-methylpyrrolidine-C12 (C_60_C12), C_60_MC12 was found to have the best performance with an electron mobility as high as 0.09 cm^2^ V^−1^ s^−1^, which is more than one order of magnitude higher than the others; furthermore, a current on/off ratio up to 4 × 10^5^ was observed. Side chains different from alkyl ones have also been used. For instance, the use of polar ethylene glycol (EG) side chains has also been explored.^14^ EG-substituted fulleropyrrolidines have been advertised as the next-generation acceptors for organic solar cells.^18^ The expectations stemmed from a predicted increased dielectric constant related to the dipoles of the polyether side chains that can rotate and align, increasing the local response to electric fields.^19–25^ Aside from the dielectric constant, the effect of the side chains on the film morphology is also substantial. Periodic dipoles easily aid crystallization, resulting in better structural order and a consequent suppressed electronic disorder in the nanomaterials.^14,21,26^ Moreover, the polarity of the EG side chains allows for good solubility and better miscibility with molecular dopants, which leads to a higher doping efficiency that enables conductivity values as high as 2.3 S cm^−1^ and the successful use of C_60_–EG derivatives as thermoelectric materials (power factor values up to 23 μW K^−2^ m^−1^).^14^ Furthermore, the high electron density of these C_60_ derivatives has been shown to be instrumental in passivating surface defects in metal halide perovskite solar cells.^27,28^

Despite the interest in C_60_–EG derivatives for different applications, their exact molecular packing remains unknown and their use in electronic devices such as transistors remains unexplored. In this work, we focus on the investigation of the structure and properties of a series of soluble C_60_ derivatives equipped with oligoethylene–glycol (EG) side chains of different lengths, namely *n* = 2 (PDEG-1), *n* = 3 (PTEG-1), *n* = 4 (PTeEG-1) and *n* = 5 (PPEG-1). All the molecules are soluble in various organic solvents, such as chloroform, and thus they are very suitable for solution processing. The transport properties of the solution-processed thin films are studied in n-channel organic field effect transistors (OFETs), showing high charge-carrier mobility values (approaching 10^−1^ cm^2^ V^−1^ s^−1^) that increase with increasing side chain length. The observed mobility increase is explained on the basis of high-resolution structural information. With a synergistic use of molecular dynamics (MD) and X-ray investigation, a clear link between the thin-film crystalline structure and order and the transport properties is established.

## Materials and method

### Materials

The synthesis of the PTEG-1 and PPEG-1 has been reported previously.^14,19^ PDEG-1 and PTeEG-1 have been synthesized following the procedure reported in the ESI.† After purification, the compounds were dried and stored in a nitrogen-filled glovebox.

### Device fabrication and characterization

Bottom-gate/bottom-contact field-effect transistors were fabricated on Si/230 nm SiO_2_ wafers carrying lithographically patterned 5 nm/35 nm ITO/Au electrodes. The fullerenes were deposited from 10 mg mL^−1^ chloroform solutions by spin coating (film thickness 40–50 nm). The devices were dried in a vacuum and measured using a Keithley 4200 Semiconductor Analyzer on a probe station inside a nitrogen-filled glovebox (<1 ppm O_2_, <1 ppm H_2_O). Multiple devices with the same geometry (20 μm × 1 cm channels) were measured to increase the certainty. Devices with a substantial gate current were excluded from the analysis. Electron mobility values were extracted from the saturation regime curves following the gradual channel approximation and assuming a parallel plate gate capacitance. The average and the range of mobilities of 2–3 devices on the same substrate were used for the discussion. The samples were stored under an inert atmosphere for the aging experiment. The exact same devices were analyzed for comparability. Samples for structural characterization were prepared by spin-coating the fullerene derivatives from chloroform solution on polished Si/SiO_2_ wafers.

### X-Ray diffraction (XRD)

XRD measurements were performed using a D8 discovery Bruker instrument. The X-ray wavelength is 0.15413 nm. Thin films of the material were measured in reflection theta–theta geometry. The probed angular range is 5° to 60° and the profiles were acquired using a 0.02 degree/step resolution.

### Grazing incidence wide angle X-ray scattering (GIWAXS)

GIWAXS measurements were performed both in the laboratory and at the European synchrotron radiation facility (ESRF), Grenoble (France). For the ESRF measurements, the X-ray wavelength was set to 0.103 nm (*E* = 12 keV). The beam size at the sample position was 300 μm. The GIWAXS patterns were acquired using a Frelon CCD camera placed 17.38 cm away from the sample and with a pixel size of 46 μm × 46 μm. The incident angle (*α*_i_) used was 0.2° and the exposure time was 1 min per frame. Images were corrected for the detector dark current and the detector efficiency (flat field correction), and the air background was subtracted. The beam center position and the angular range were calibrated using the known position of diffraction peaks from a standard silver behenate sample.

For the laboratory, GIWAXS measurements were acquired at the MINA instrument in Groningen. The instrument is built on a high flux rotating anode X-ray source (wavelength of 0.15413 nm, *E* = 8 keV). The patterns were acquired using a Vantec500 Bruker detector placed 10 cm away from the sample and with a pixel size of 136 μm × 136 μm. The incident angle (*α*_i_) used was 0.15° and the exposure time was 30 min per frame. Images were corrected for detector geometrical distortion and detector efficiency (flat field correction). The beam center position and the angular range were calibrated using the known position of diffraction peaks from a standard silver behenate sample.

GIWAXS patterns are plotted against the horizontal *q*_*y*_ and quasi-vertical *q*_*z*_ scattering vector scale, where
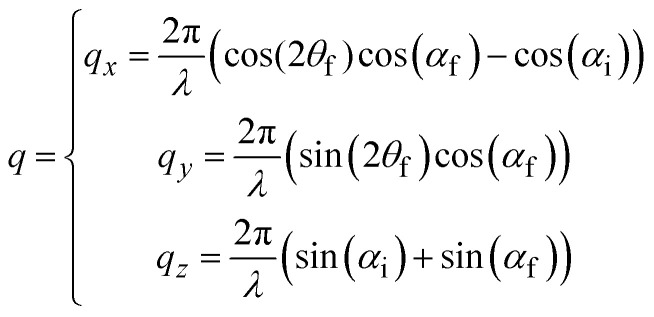
GIWAXS patterns were further analyzed using the GIXSGUI Matlab package.^29^

### Molecular dynamics

The force fields necessary for the MD simulations were adapted from the non-polarizable PTEG-1 force field recently developed by Sami *et al.*^24^ Briefly, the force field uses Lennard-Jones (LJ) parameters from the GROMOS 54A6 parameter set,^30^ based on previous work,^31^ which is improved by the derivation of bonded parameters (bond, angle, and dihedral parameters) from quantum chemical calculations, following the Q-Force procedure.^32,33^ Force fields for the remainder of the C_60_–EG series were generated by adding or removing EG chains from the PTEG-1 force field.

Using the layered structure indicated by the GIWAXS data as a basis, MD simulations were performed in order to obtain atomistic configurations for the two molecules in a tetragonal unit cell, as similarly done recently for another fullerene derivative.^26^ For the body-centered tetragonal unit cell (the PDEG-1 case), the 2-molecule unit cell was duplicated in the *c* direction to have 4 molecules. Periodic boundary conditions were applied in three directions. The MD simulations were carried out in several steps to maximize sampling. The three unit-cell parameters, *a*, *b*, and *c*, were allowed to fully relax during the MD simulations. The simulations were repeated 360 or 720 times until the mean of the lattice cell parameters reached the desired convergence (standard error < 0.002 nm). Diffraction patterns were computed using Dans_Diffraction.^34^ Additional simulation details can be found in the ESI.†

## Results and discussions

**Fig. 1:**
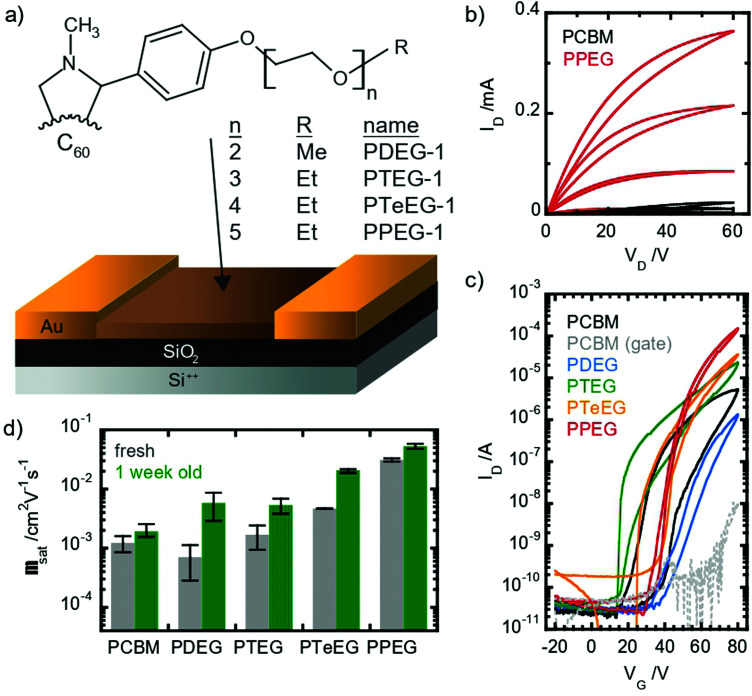
Transport properties in thin films of fullerene derivatives with different side-chain lengths. (a) Molecular structure of the studied materials: R = CH_3_ for PDEG-1 and CH_2_CH_3_ for all the other samples; (b) output curves measured in as-prepared PCBM and PPEG-1 transistors in a saturation regime showing a unipolar n-type characteristic and molecule-dependent conductance; (c) comparison of the transfer characteristics measured in the as-prepared OFETs using the different materials; and (d) mobility values extracted from the as-prepared and aged devices, where the error bars represent one standard deviation.

OFETs have been prepared using four different soluble C_60_ derivatives equipped with pendant EG chains (C_60_–EG) of different lengths (*n* = 2 for PDEG-1, 3 for PTEG-1, 4 for PTeEG-1 and 5 for PPEG-1, where *n* is the number of EG units; see Fig. 1a). Moreover, a transistor using a PCBM thin film was also prepared for comparison, as PCBM has been extensively studied in OFETs as an n-type material.^35–39^

All devices showed unipolar n-type behavior: clear linear and saturation regimes are observed in the output curves in the first quadrant (Fig. 1b). Large differences between the different molecules are displayed by the IV characteristic. Somehow counterintuitively, the chain length seems to have an inverse effect on the saturation current: the longer the chain, the higher the current (see Fig. 1c). The hysteresis appears to be lower in the EG-functionalized materials compared with PCBM, especially in the PTeEG-1 and PPEG-1 devices (Fig. 1c).

Electron mobility values were extracted from the saturation regime curves following the gradual channel approximation and assuming a parallel plate gate capacitance. The plotted values are shown in Fig. 1d (the data points represent the average, and the error bar derives from a range of 2–3 nominally identical devices); the mobility increases with the side chain length, as expected from the increasing current. The side chain engineering leads to average mobilities spanning a two-orders-of-magnitude range. The 2- and 3 unit side chain molecules show a similar mobility to the PCBM reference, namely, around 10^−3^ cm^2^ V^−1^ s^−1^, while the values for PTeEG-1 (4 unit) and PPEG-1 (5 unit) approach and exceed 10^−2^ cm^2^ V^−1^ s^−1^.

When comparing the mobility of freshly prepared and one-week-aged samples (see Fig. 1d), we found that the mobility increases upon aging, suggesting a coarsening of the thin films over time. The coarsening hypothesis is confirmed by performing grazing incident wide-angle X-ray scattering (GIWAXS) measurements (Fig. S1, ESI†). After deposition, the PPEG-1 film is observed to be crystalline and shows good crystallite alignment, but the off-specular reflections (*q*_*y*_ and *q*_*z*_ ≠ 0) appear to be quite broad, suggesting small average crystallite dimensions. After one week of storage in an inert atmosphere, the thin film undergoes an increase in the crystallite size (coarsening) as can be inferred by the sharpening of the diffracted off-specular spots. A more refined structure with a larger crystallite size gives rise to lower electronic disorder and increased intermolecular coupling, enabling the higher charge mobility.

Given the lack of chemical differences between the fulleropyrrolidine series, the differences in the transport properties can only be explained by structural factors. Film crystallinity plays an important role in the quality of transport in fullerene derivatives,^40–42^ and thus can account for the observed changes. Here, it is important to underline that the thin films were fabricated using chloroform and we expect that even higher mobilities can be obtained using solvents with a lower vapor pressure, which could favor the crystallization process.

**Fig. 2:**
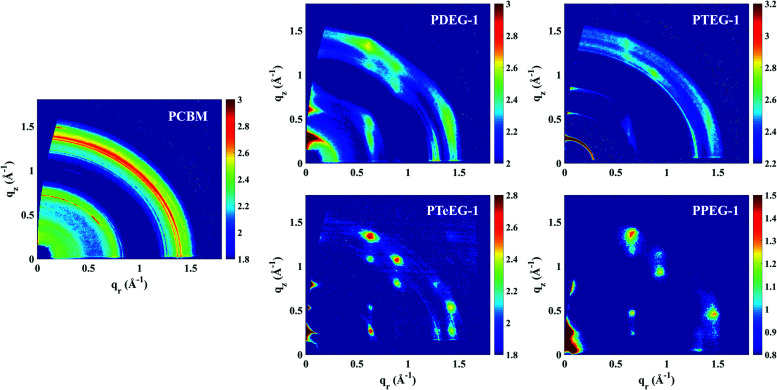
GIWAXS patterns for the C_60_–EG derivatives spin coated from chloroform solutions. The GIWAXS for a PCBM thin film is also reported for comparison. Note that the PPEG-1 sample was measured using the lab X-ray instrument, while all the others were measured at the synchrotron.

In order to study the molecular packing, crystallinity and crystallite orientation within the C_60_–EG thin films, a full GIWAXS study was performed. Fig. 2 shows the GIWAXS patterns for the thin films deposited on the Si/SiO_2_ substrate and acquired using an incident angle of *α*_i_ = 0.2°.

All the C_60_–EG thin films show crystalline behavior as evidenced by the presence of sharp diffraction signals in the GIWAXS patterns. Nevertheless, some differences can be highlighted. For instance, the angular spreading of the GIWAXS signals changes dramatically between the different samples. We reiterate that the nature and the angular spreading of the GIWAXS signals are indicative of the sample morphology and crystallite orientation. Thin films composed of highly oriented crystallites show well-defined diffraction spots, while thin films composed of isotopically oriented powder-like crystallites exhibit homogeneously distributed Debye–Scherrer diffraction rings. The PCBM thin film hardly shows any anisotropic signals and the GIWAXS pattern is dominated by Debye–Scherrer rings, indicating the polycrystalline nature of the film, where the crystallites are randomly oriented with respect to the substrate. Remarkably, the addition of the EG side chains induces a different structure and provides the system with the ability to spontaneously assemble during spin coating with the preferential alignment of the crystallites with respect to the substrate surface.

The PDEG-1 and PTEG-1 films show diffraction arcs, rather spread along the azimuthal angle, especially for PTEG-1, indicating a lower degree of orientation. Moreover, in the GIWAXS patterns of the PDEG-1 and PTEG-1 films, the diffraction arcs partially overlap with isotropic diffracted rings, suggesting that a certain portion of the crystallites is randomly aligned through the films. On the contrary, discrete diffraction spots appear for the molecules with the longest EG chains, namely PTeEG-1 and PPEG-1.

**Fig. 3:**
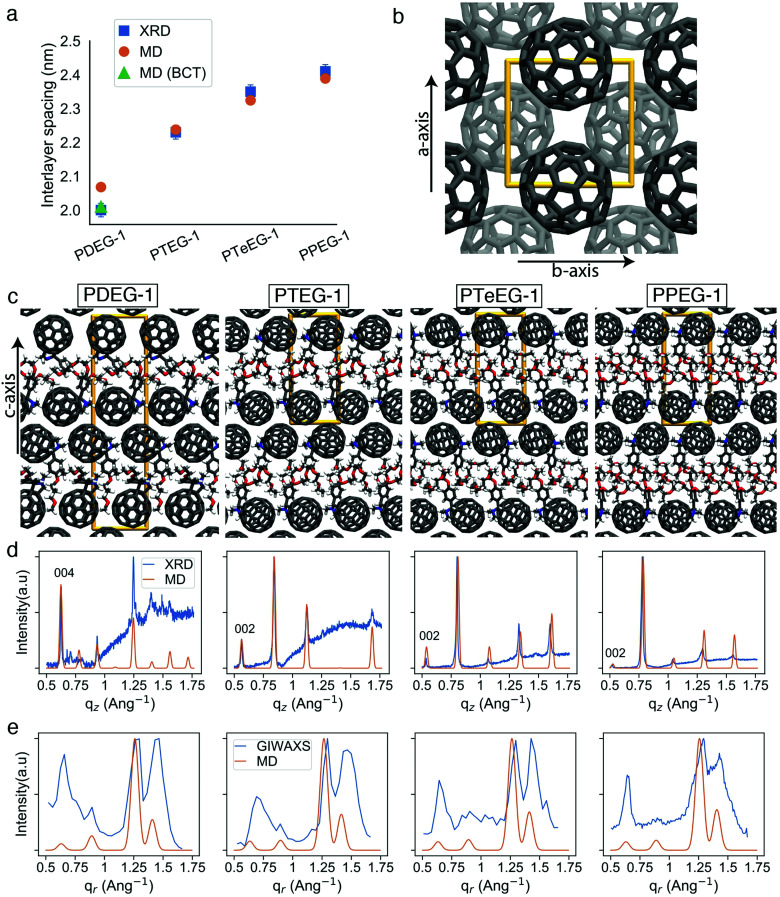
Comparison of X-ray and MD characterization of C_60_–EG derivative thin films. (a) Interlayer spacing extracted from the XRD data and obtained from the MD simulations. Orange circles correspond to the interlayer spacing of tetragonal primitive unit cells (2 molecules in the unit cell) and the green triangle corresponds to the interlayer spacing (half of the *c* lattice parameter) for a body-centered tetragonal (BCT) unit cell (4 molecules in the unit cell); (b) representative view of the C_60_ molecules along the *c* axis, other atoms not shown (similar for all molecules); (c) representative structures for the C_60_–EG series from the MD simulations along the *a* or *b* axis; (d) experimental thin film XRD and MD simulated diffraction patterns corresponding to the representative unit cells from (c); and (e) experimental GIWAXS and MD simulated patterns along the *q*_r_ direction.

Several high intensity reflections are observed along the vertical *q*_*z*_ direction perpendicular to the substrate both in the GIWAXS patterns (Fig. 2) and in the thin film XRD profiles (Fig. 3d). The positions of these reflections are q*, 2q*, 3q* *etc.*, suggesting a layer-like arrangement of the molecules with the 00*l* diffraction planes parallel to the substrate surface. The interlayer spacing can be calculated as 2π/q*. All the GIWAXS patterns except the for PDEG-1 can be indexed using a tetragonal primitive unit cell of axis *a* × *b* × *c* (see Fig. S2 and Table S1, ESI†). In the case of PDEG-1, the GIWAXS pattern is better indexed using a body-centered tetragonal unit cell. We found that *a* = *b* ≈ 1 nm for all the samples, independent of the EG chain length. Considering that the C_60_ diameter is 1 nm, this means that a strong π–π overlap between the C_60_ units must exist. By contrast with *a* and *b*, the value of the interlayer spacing (and thus the *c* axis) varies with the number of EG units in the pendant chain, as reported in Fig. 3a. Such a layered structure made by alternating fullerene and EG layers is expected on the basis of a spontaneous phase separation between the fullerene moieties and the polar –CH_2_CH_2_O–chains. A similar layered structure has been observed for long-chain alkyl-substituted C_60_.^16^ The confinement of the C_60_ molecules in discrete, well-aligned layers is responsible for the good transport features discussed above and for the observed improved charge transport when compared with PCBM.

The evolution of the interlayer spacing with the number of EG units in the side chain (reported in Fig. 3a as blue squares) shows a close to linear growth of about 0.1 nm per EG unit. This value is much smaller than the length of a single EG unit (∼0.3–0.4 nm). Thus, EG chain interdigitation, bending, and coiling are expected. The interlayer spacing measured for PTeEG-1 of 2.35 nm is comparable to that of 2.32 nm reported for the alkyl-substituted C_60_ derivative with a dodecyl side chain, as the two side chains are of comparable length.^16^ In this case, ordered interdigitating of the dodecyl side chains was reported. It is interesting to note how the structure of these thin films differs from the one adopted by the same materials in single bilayers.^43^ For instance, PTEG-1 bilayers have been reported to have a fullerene–fullerene distance of 3.3 nm, sensibly larger than the 2.23 nm found here for the multilayers in the thin films.

Unfortunately, little information can be retrieved from the X-ray data on the arrangement and packing of the fullerene moieties and the EG side chains and on the overall atomic positions in the unit cell for these thin films. In order to learn more about the side chain packing and to resolve the layered nature of the C_60_–EG thin film structure, atomistic MD simulations were performed. The number of molecules in the unit cell and the approximate relative size of the lattice parameters were inferred from the XRD data (Fig. 3a) and were used as the starting point for the MD simulations. Both lattice parameters (*a*, *b*, *c*) and atomic positions were then fully relaxed and more than 300 different realizations of the unit cells were generated for each molecule. Further information on the procedure can be found in the Methods section and the ESI.†

Inspection of the simulated structures provides immediate support for the layered structure inferred by GIWAXS for all of the fulleropyrrolidine derivatives (see Fig. 3c). The close staggered arrangement of the fullerene moieties observed in the MD simulations results in an in-plane distance of ∼1 nm between the fullerene moieties for all the molecules (see Fig. 3b). This value is in excellent agreement with the GIWAXS data reported in Table S1 (ESI†). The close proximity of the fullerene moieties also results in a significant π–π orbital overlap, responsible for the good mobility values exhibited by these molecules. Approximately, the size of the C_60_ phase in the *c* axis is 1.4 nm and the EG phase grows from 0.6 nm to 0.8, 0.9 and 1.0 nm for PDEG-1, PTEG-1, PTeEG-1, PPEG-1, respectively. Consequently, the interlayer spacing also grows accordingly.

Comparison of the simulated interlayer spacing shows excellent agreement with the XRD data. For *n* = 3–5 samples, the interlayer spacing of the primitive tetragonal two-molecule unit cells obtained from MD simulations (orange circles in Fig. 3a) is in perfect agreement with the XRD data while *n* = 2 (PDEG-1) overestimates it. However, using a body-centered tetragonal (BCT) four-molecule unit cell that is replicated in the *c* direction (green triangle in Fig. 3a) results in a smaller interlayer spacing that is in agreement with XRD, indicating that better packing was possible due to the additional freedom provided by the larger unit cell. This enforces the better suitability of the BCT unit cell for PDEG-1, as has also been shown based on the GIWAXS and simulated diffraction patterns of the {10*l*} family of planes (Fig. S2, ESI†).

**Fig. 4:**
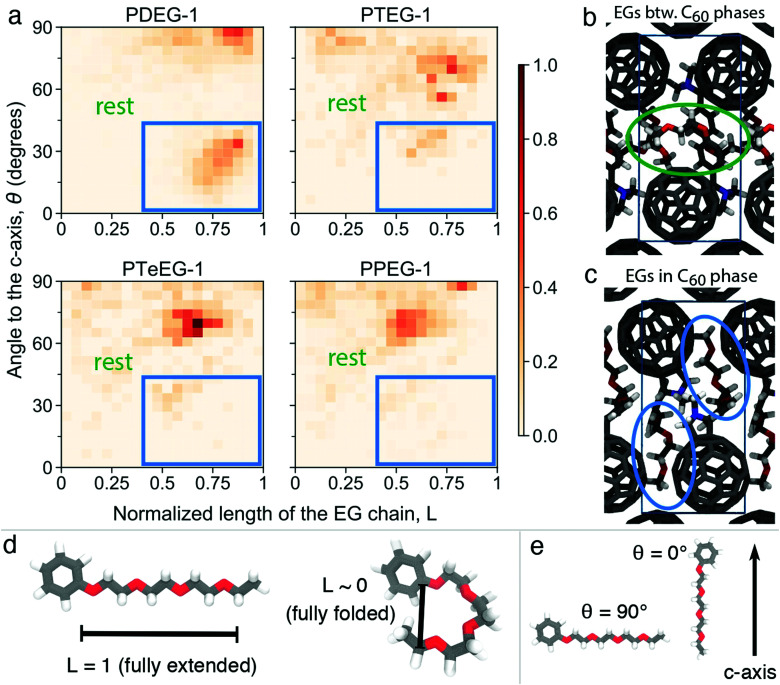
Structural analysis of the unit cells of C_60_–EG derivatives from atomistic MD simulations. (a) 2D histogram of the normalized length of the EG chains (*L*) against the angle (*θ*) between the direction of the EG chain (*L* vector) and the *c* axis for all molecules obtained from the MD simulations. *L* is normalized, as also depicted in (d), so that it corresponds to 1 for the fully extended case and to 0 for the fully folded case (0.34 nm, based on van der Waals radii). For *θ*, as also depicted in (e), 0° means that the EG chain is aligned parallel to the *c* axis, leading to a chain-end that enters the C_60_ phase with a large enough *L* (depicted in (c)), and 90° means that the EG chain is aligned perpendicular to the *c* axis, leading to an EG that remains in between the two C_60_ phases (depicted in (b)). The histograms are normalized together, meaning that the highest occurrence (the darkest color) corresponds to the one among all molecules.

Furthermore, structural information, namely the arrangement of the EG chains for the C_60_–EG series, can also be extracted from the MD simulations. The flexible nature of the EG chains provided by the low torsional barrier results in an ensemble of similar configurations instead of a unique one.^24^ Consequently, in order to analyze their arrangement, the distribution of two descriptors among all realizations is investigated (see Fig. 4a). The first descriptor is the normalized length of the EG chain (*L*) that gives 1 for the fully extended side chain and 0 for the fully folded one (see Fig. 4d for their depiction). The second one is the angle (*θ*) between the direction of the EG chain (*L* vector) and the *c* axis (see Fig. 4e for their depiction) that gives 0° when the chains are aligned parallel to the *c* axis and 90° when they are aligned perpendicularly. Regarding the extension of the EG chains (*L*), the large majority of the realizations for all molecules have 0.4 < *L* < 0.8, meaning that the chains are partially extended but almost never fully extended. Regarding the angle with respect to the *c* axis (*θ*), *θ* > 45° becomes more prominent as the number of EG units increases, meaning that they are closer to a perpendicular arrangement. In particular, PDEG-1 has a large number of configurations with *θ* < 45°. Combination of a partially extended side chain (*L* > 0.4) together with a near parallel alignment of the side chain with the *c* axis (*θ* < 45°) leads to penetration of the EGs into the C_60_ phase (depicted in Fig. 4c, corresponding to the blue circled region). In all other cases, EGs remain between the two C_60_ phases, forming an EG phase (depicted in Fig. 4b). This penetration, calculated from the distance of EG chain ends to the fullerenes in the *c* axis (see Fig. S4, ESI†), occurs much more prominently for the shorter side chain molecules (46% and 26% for PDEG-1 and PTEG-1, respectively) compared with the longer side chain molecules (18% and 12% for PTeEG-1 and PPEG-1, respectively). In addition, the average distance between the center of mass of fullerenes has been computed (Table S3, ESI†). This distance does not depend on the side chain length, in agreement with the very similar spacing of the unit cell in the *a* and *b* directions (see Table S1, ESI†), which is determined by the size of C_60_ and the C_60_–C_60_ distance. Finally, the molecules with the longer EG chains show the narrowest distribution of configurations, while PDEG-1 and PTEG-1 appear to have the broadest, indicating an increased tendency for structural disorder when EG is too short, in agreement with the presence of randomly aligned crystallites for the shorter EG chains, as observed by GIWAXS (Fig. 2).

Theoretical structural predictions appear to align well with the experimentally observed structural and transport property trends for the C_60_–EG series reported here (Fig. 1) and also with previous data published by Liu *et al.*^14^ Interestingly, the increasing electron mobility with the increase of *n* also agrees with the growing size of the EG phase predicted by the MD simulations. This suggests that a thicker EG phase could improve the in-plane mobility by reducing the Coulombic interaction of charge carriers between different C_60_ layers due to increased spacing. Additionally, the higher penetration of the EG molecules in the C_60_ phase for the shorter EG chain molecules, as shown in Fig. 4, may also be responsible for the lower charge transport, as these penetrating EG molecules would hinder the transport between the fullerenes.

## Conclusions

In this work, we have studied the transport properties and the structure of thin films of fullerene derivatives with oligoethylene–glycol side chains (C_60_–EG) of different lengths, from *n* = 2 to *n* = 5, where *n* is the number of EG units in the side chain. Field effect transistors have been successfully prepared using the full series of fullerene derivatives and they show high values of mobility that increases with increasing the side chain length. The observed increase in mobility was explained here on the basis of high-resolution structural information obtained through the synergistic use of experimental X-ray data and MD computational methods.

X-ray structural results reinforced by MD simulations have revealed that the growth of the interlayer spacing with the number of EG units in the side chain, *n*, corresponds to the growth of the EG layer, where the EG chains are splayed rather than perpendicular to the fullerene layers. As a result of these layered structures, fullerene moieties pack closely and form efficient electron-transport layers that are oriented parallel to the substrate surface. Increasing the number of EG units above 3 causes an increase in the molecular packing and crystallite orientation, boosting the mobility up to an average value of 0.053 cm^2^ V^−1^ s^−1^ for the best device with PPEG-1 (*n* = 5), which is more than one order of magnitude higher than the value exhibited by PCBM in this study.

The synergistic use of high-resolution XRD and advanced GIWAXS methods together with MD simulations seems to be a perfect tool to understand the transport properties of fullerene-based thin film devices and can be used to drive the chemical design of fullerene derivatives in the future.

## Author contributions

G. P. designed and supervised research. J. D. performed the GIWAXS measurements under the supervision of G. P. The computational experiments were performed by S. S., and S. S. and R. A. devised the computational protocols; both of these were done under the supervision of S. J. M., R. W. A. H and G. P. The electrical measurements were performed by D. M. B. under the supervision of M. A. L. The materials were synthesized by F. J. and L. Q. under the supervision of J. C. H. The data were analyzed by J. D., S. S., D. M. B., R. A., R. W. A. H. and G. P. The manuscript was conceived and written by J. D., S. S., M. A. L., and G. P. All the authors read and approved the manuscript before submission.

## Conflicts of interest

The authors declare that they have no competing interests.

## Supplementary Material

TC-009-D1TC02753K-s001

TC-009-D1TC02753K-s002

TC-009-D1TC02753K-s003

TC-009-D1TC02753K-s004

TC-009-D1TC02753K-s005

TC-009-D1TC02753K-s006

TC-009-D1TC02753K-s007

TC-009-D1TC02753K-s008

TC-009-D1TC02753K-s009

TC-009-D1TC02753K-s010

TC-009-D1TC02753K-s011

TC-009-D1TC02753K-s012

TC-009-D1TC02753K-s013

TC-009-D1TC02753K-s014

TC-009-D1TC02753K-s015

TC-009-D1TC02753K-s016

TC-009-D1TC02753K-s017

TC-009-D1TC02753K-s018

TC-009-D1TC02753K-s019

TC-009-D1TC02753K-s020

TC-009-D1TC02753K-s021

TC-009-D1TC02753K-s022
