# Supplementary material for: Fullerene derivatives with oligoethylene–glycol side chains: an investigation on the origin of their outstanding transport properties
Source: J Mater Chem C Mater. 2021 Aug 18;9(45):16217–25. doi: 10.1039/d1tc02753k (PMC8614456; doi:10.1039/d1tc02753k)
Supplement: TC-009-D1TC02753K-s001 [file TC-009-D1TC02753K-s001.pdf]

Electronic Supplementary Material (ESI) for Journal of Materials Chemistry C.
This journal is © The Royal Society of Chemistry 2021

Fullerene derivatives with oligoethylene-glycol side chains: an investigation on the origin of their outstanding transport properties

Jingjin Dong[†1], Selim Sami[†1,2,3], Daniel M. Balazs[1#], Riccardo. Alessandri[1,3§], Fatimeh Jahani[2], Li Qiu[2], Siewert J. Marrink[1,3], Remco W.A. Havenith[1,2,4], Jan C. Hummelen[1,2], Maria A. Loi[1], G. Portale[1*]

[1] Zernike Institute for Advanced Materials, University of Groningen, Nijenborgh 4, Groningen, 9747AG, the Netherlands

[2] Stratingh Institute for Chemistry, University of Groningen, Nijenborgh 4, 9747 AG Groningen, The Netherlands

[3] Groningen Biomolecular Sciences and Biotechnology Institute, University of Groningen, Nijenborgh 7, Groningen, 9747AG, The Netherlands

[4] Department of Chemistry, Ghent University, Krijgslaan 281 (S3), B-9000 Gent, Belgium

# Current address: [#] IST Austria, Am Campus 1, 3400, Klosterneuburg, Austria

§ Current address: Pritzker School of Molecular Engineering, University of Chicago, Chicago, IL 60637, USA

[†] Equal contribution

Email: g.portale@rug.nl

SUPPORTING INFORMATION

Synthesis of the PDEG-1 and PTeEG-1 compounds

The C_{60} used for the syntheses was of 99.5% purity (purchased from Solenne BV, Groningen, The Netherlands). Analytical thin-layer chromatography (TLC) was performed on Merck silica gel 60/kieselguhr F254, and visualization was accomplished by UV light. Column chromatography was performed using silica gel (SiliaFlash P60 Type R12030B, 230-400 mesh). ^{1}H-NMR and ^{13}C-NMR were performed on a Varian Unity Plus (400 MHz) instrument at 25 °C, using tetramethylsilane (TMS) as an internal standard. NMR shifts are reported in ppm, relative to the residual protonated solvent signals of $CDCl_3$ (δ = 7.26 ppm) or at the carbon absorption in $CDCl_3$ (δ = 77.0 ppm), and multiplicities are denoted as s = singlet, d = doublet, t = triplet, m = multiplet and b = broad. IR measurements were performed on a Nicolet iS50 FT-IR spectrometer. High-resolution mass spectroscopy (HRMS) was performed on a JEOL JMS 600 spectrometer.

PTeEG-1:

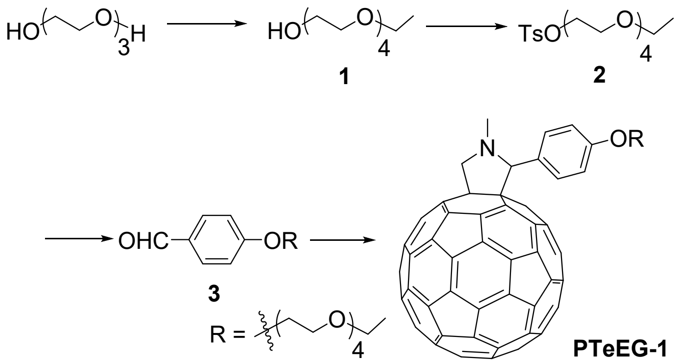

(a) Synthesis of tetraethylene glycol monoethyl ether **1**: Triethylene glycol (2.25 g, 15 mmol) in 30 mL of anhydrous THF was added dropwise to a suspension of NaH (60% dispersion in mineral oil) (0.78 g, 19.5 mmol) in 15 mL of anhydrous THF at 0 °C. This mixture was stirred for a further 1 h at 0 °C, and then, a solution of 2-ethoxyethyl 4-methylbenzenesulfonate (3.6 g, 15 mmol) in 15 mL of THF was added dropwise. This mixture was allowed to warm to rt for 1 h and then heated to reflux for 12 h. The reaction mixture was cooled to rt and filtered, and all volatile materials were removed by rotary evaporation. The yellow oil was dissolved in toluene (25 mL), and the organic layer was extracted with water. The aqueous layer was extracted with dichloromethane, and the combined organic layer was dried with Na_2SO_4; the solvent was then removed by rotary evaporation. The yellow oil obtained was purified by column chromatography (silica gel, dichloromethane/ethyl acetate 3:1 to 1:1) to give the desired compound **1** as a light yellow oil (2.4 g, 72%).

(b) Synthesis of the tosylate of tetraethylene glycol monoethyl ether **2**: Sodium hydroxide (0.16 g, 4 mmol) dissolved in water (1 mL) and compound **1** (0.6 g, 2.7 mmol) in THF (2 mL) were placed in a three-necked, 25 mL round-bottom flask. The mixture was cooled on an ice bath. *p*-Toluenesulfonyl chloride (0.48 g, 2.6 mmol) in THF (3 mL) was added dropwise to the mixture. The solution was stirred at 0 °C for an additional 3 h and then poured into ice-water (20 mL) and extracted with ethyl acetate. The organic layer was washed with water and dried over

Na_2SO_4. The solvent was evaporated *in vacuo*. The crude product obtained was used directly in the next step (0.8 g, 82%).

(c) Synthesis of 'tegylated' benzaldehyde **3**: A three-necked, 250 mL round-bottom flask was charged with *p*-hydroxybenzaldehyde (0.24 g, 1.93 mmol), compound **2** (0.8 g, 2.12 mmol), K_2CO_3 (0.8 g, 5.8 mmol) and DMF (10 mL). The reaction mixture was stirred overnight at 90 °C. After cooling, the crude reaction mixture was poured into water (100 mL, pH = 2) and extracted with ethyl acetate. The organic layer was washed subsequently with water (3 x 25 mL) and brine (1 x 25 mL) and dried over Na_2SO_4. The solvent was evaporated *in vacuo*. The crude light yellow oil **3** was pure enough to be used directly in the next step (0.49 g, 78%).
^{1}H NMR (400 MHz, $CDCl_3$) δ 9.88 (s, 1H), 7.82 (d, *J* = 8.8 Hz, 2H), 7.02 (d, *J* = 8.7 Hz, 2H), 4.25–4.16 (m, 2H), 3.95–3.85 (m, 2H), 3.72 (d, *J* = 5.5 Hz, 2H), 3.65 (d, *J* = 8.1 Hz, 8H), 3.57 (s, 2H), 3.51 (q, *J* = 7.0 Hz, 2H), 1.20 (t, *J* = 7.0 Hz, 3H). ^{13}C NMR (100 MHz, $CDCl_3$) δ 193.4, 166.5, 134.6, 132.7, 117.5, 73.6, 73.3, 72.5, 72.1, 70.4, 69.3, 17.8. IR (cm^{-1}): 3553, 2865, 1685, 1598, 1574, 1507, 1254, 1101, 1051, 829.

(d) Synthesis of **PTeEG-1**. An oven-dried three-necked, 250 mL round-bottom flask was charged with C_{60} (1.08 g, 1.5 mmol), compound **3** (1.6 mmol), sarcosine (0.44 g, 4.9 mmol) and *o*-dichlorobenzene (100 mL). The reaction mixture was stirred under N_2 at 90 °C for 72 h. The mixture was concentrated *in vacuo* to 15 mL, and the crude residue was purified by column chromatography (silica gel; toluene/ethyl acetate 4:1) to afford the pure compound as brown solid. The product was redissolved in 7 mL of chlorobenzene, precipitated with MeOH, washed repeatedly with MeOH and pentane, and dried in vacuo at 50 °C. This procedure gave pure **PTeEG-1** (36%)).
^{1}H NMR (400 MHz, $CDCl_3$) δ 7.69 (s, 2H), 6.96 (d, *J* = 8.4 Hz, 2H), 4.93 (d, *J* = 31.7 Hz, 2H), 4.25 (d, *J* = 8.5 Hz, 1H), 4.17–4.09 (m, 2H), 3.91–3.77 (m, 2H), 3.73–3.55 (m, 12H), 3.50 (q, *J* = 7.0 Hz, 2H), 2.79 (s, 3H), 1.19 (t, *J* = 7.0 Hz, 3H). ^{13}C NMR (100 MHz, $CDCl_3$) δ 158.9, 157.9, 153.5, 147.3, 146.3, 146.2, 146.1, 146.0, 145.8, 145.5, 145.3, 145.2, 145.1, 144.7, 144.6, 144.4, 143.1, 143.0, 142.7, 142.6, 142.5, 142.2, 142.0, 141.9, 141.7, 141.5, 140.1, 140.0, 139.6, 136.9, 136.6, 135.8, 130.5, 114.7, 83.1, 70.8, 70.7, 70.6, 69.8, 69.7, 68.9, 67.3, 66.6, 40.0, 15.2. IR (cm^{-1}): 2862, 2777, 2328, 1609, 1509, 1456, 1249, 1110, 1062, 927, 765. HRMS(ESI) calcd. for $C_{79}H_{32}NO_5[M+H]^+$: 1074.22750, found: 1074.22795.

PDEG-1
When using 4-(2-(2-methoxyethoxy)ethoxy)benzaldehyde instead of compound **3** as the substrate, the above procedure gave **PDEG-1** (40%) as a brown solid.
^{1}H NMR (400 MHz, $CDCl_3$) δ 7.69 (s, 2H), 6.96 (d, *J* = 8.7 Hz, 2H), 4.97 (d, *J* = 9.4 Hz, 1H), 4.87 (s, 1H), 4.23 (d, *J* = 9.4 Hz, 1H), 4.19–4.09 (m, 2H), 3.91–3.80 (m, 2H), 3.71 (dd, *J* = 5.5, 3.7 Hz, 2H), 3.60–3.51 (m, 2H), 3.37 (s, 3H), 2.78 (s, 3H).
^{13}C NMR (100 MHz, $CDCl_3$) δ 158.8, 156.4, 154.1, 153.6, 147.3, 146.8, 146.5, 146.3, 146.2, 146.1, 146.0, 145.8, 145.5, 145.3, 145.2, 145.1, 144.7, 144.6, 144.4, 143.1, 143.0, 142.7, 142.6, 142.5, 142.3, 142.2, 142.1, 142.0, 141.8, 141.7, 141.5, 140.1, 139.9, 139.6, 136.8, 136.5, 135.8, 130.4, 129.1, 114.7, 83.2, 71.9, 70.8, 70.0, 69.8, 69.0, 67.3, 59.1, 40.0.

IR (cm^{-1}): 2945, 2871, 2775, 2328, 1610, 1510, 1463, 1426, 1330, 1246, 1173, 1122, 1029, 840.
HRMS(ESI) calcd. for $C_{74}H_{22}NO_3[M+H]^+$: 972.15942, found: 972.15853.
The relevant NMR spectra are attached in the end of SI, as Figure S5-S10.

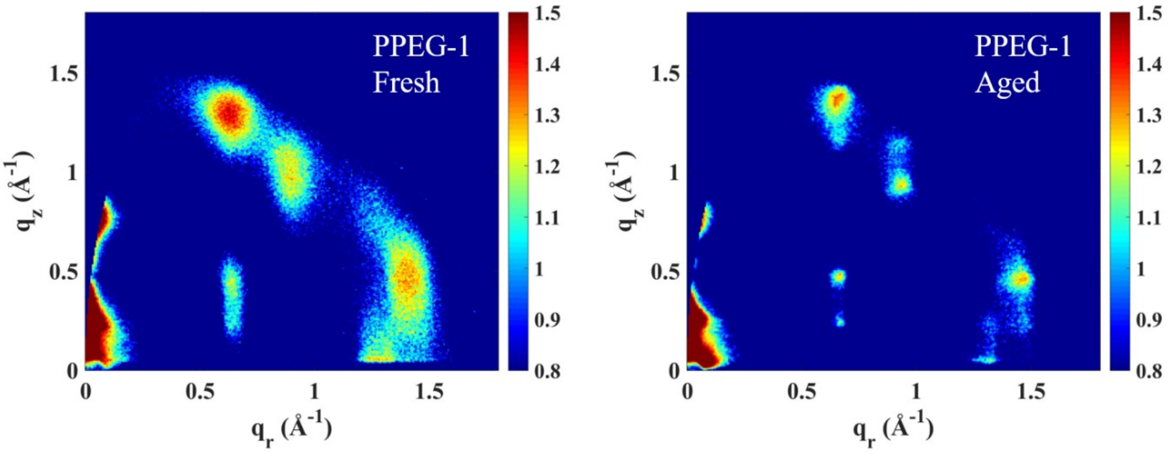

Fig. S1 GIWAXS patters for a PPEG-1 film (n = 5) freshly prepared and for the same film aged for 1 week. These patterns were acquired using a lab X-ray instrument.

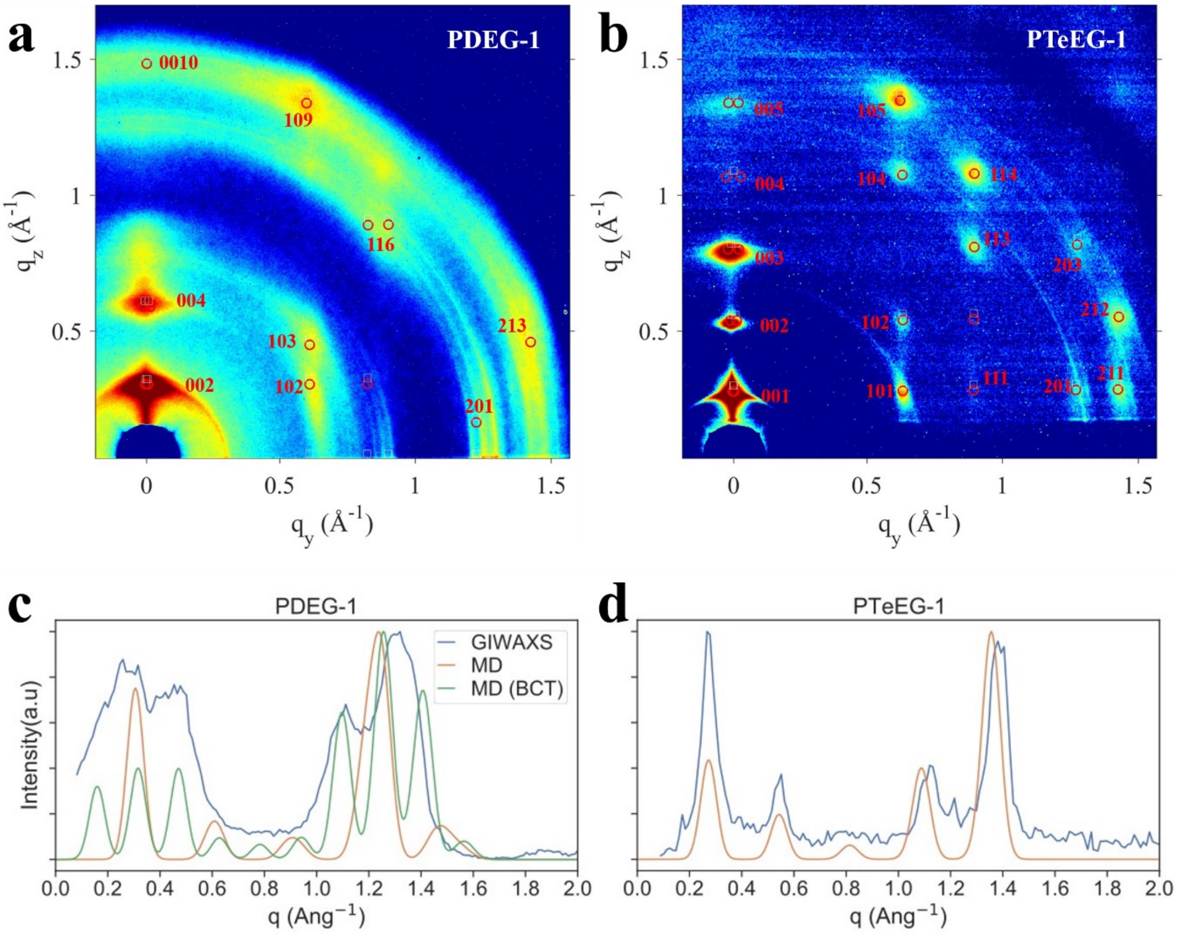

Fig. S2 a) and b) GIWAXS peak assignment using a body-centered tetragonal (BCT) unit cell for the PDEG-1 sample and a primitive tetragonal unit cell for the PTeEG-1. c-d) comparison between the GIWAXS line cuts and the MD simulated profiles (orange curve for a primitive tetragonal unit cell and green curve for a BCT unit cell) along the [10l] direction.

Table S1 Unit cell parameters for the C_{60}-EG thin films as determined from the GIWAXS data

	a	b	c (nm)		Angle
PDEG-1	1.00	1.00	4.0	Tetragonal (I 4/m)	(90°,90°,90°)
PTEG-1	1.00	1.00	2.23	Tetragonal (P 4)	(90°,90°,90°)
PTeEG-1	0.98	0.98	2.35	Tetragonal (P 4)	(90°,90°,90°)
PPEG-1	0.98	0.98	2.41	Tetragonal (P 4)	(90°,90°,90°)

Table S2 Relative Crystallinity (RC) for the C_{60}-EG thin films as determined by the analysis of the 001 GIWAXS reflection. RC was deduced from I(χ) polar plots at *001* peak, χ being the polar angle, and the area below the I(χ) × sin(χ) versus χ plots (A_c) were obtained by full-integration between 0 and 90°.[1] The one of PDEG-1 is normalized to 1.

	PDEG-1	PTEG-1	PTeG-1	PPEG-1
RC	1 (A_c = 112 K)	1.38	3.77	7.28

Computational Methods

The following 4 MD simulation steps were carried out in series on starting configurations with unit cell dimensions of 2 x 2 x 4.5 nm^3: 1) 2000 K, flat-bottom restraint, 2 ns; 2) 2000 K, 2 ns; 3) gradual cooling from 2000 to 298 K over 2 ns; 4) 298 K, 2 ns. This leads to a total of 8 ns of MD simulations (2 ns per step). In the case of the body centered unit cell: 1) A snapshot taken from previous step 3 at 1000K and the unit cell is doubled in c direction; 2) annealed at 1000K for 2 ns; 3) gradually cooled to 298K over 2 ns; 4) relaxed at 298K for 2 ns. Due to the small size of the unit cell, shorter cutoffs were used for LJ and Coulomb interactions (0.45 nm). However, particle mesh Ewald (PME) method was used for both LJ and Coulomb interactions to account for interactions beyond this cutoff.[2] Weak coupling schemes were used for both temperature and pressure.[3] The pressure was maintained at 1 bar anisotropically for the three lattice cell parameters, *a*, *b*, and *c* with a compressibility of $5x10^{-6}$ bar^{-1}. Coupling parameters were 1.0 and 0.5 ps for temperature and pressure, respectively. The flat-bottom potential during the first step kept the two C_{60} moieties at the top and bottom of the unit cell with respect to the *c* axis based in order to keep the *c*-axis spacing the longer one, in accordance with experimental X-ray measurements. The MD simulations were performed using the GROMACS 2018.5 software package.[4] Following the protocol above, 720, 360, 360, and 720 independent MD simulations were run on PDEG-1, PTEG-1, PTeEG-1, and PPEG-1, respectively. Results presented are either distribution or the mean of these independent runs, for each molecule. Interlayer spacing distributions for the C_{60}-EG series are given in Figure S3. For the simulated scattering, Gaussian broadening with standard deviations of 0.01 Ang^{-1} and 0.03 Ang^{-1} were used in figures corresponding to XRD and GIWAXS, respectively. Files to reproduce the computational work are provided as part of the SI files.

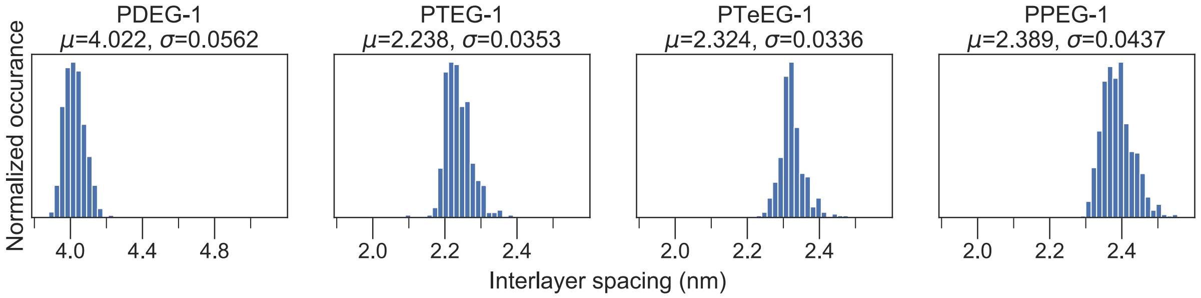

Fig. S3 Distribution of the interlayer spacing together. The mean (μ) and the standard deviation (σ) is given for all molecules.

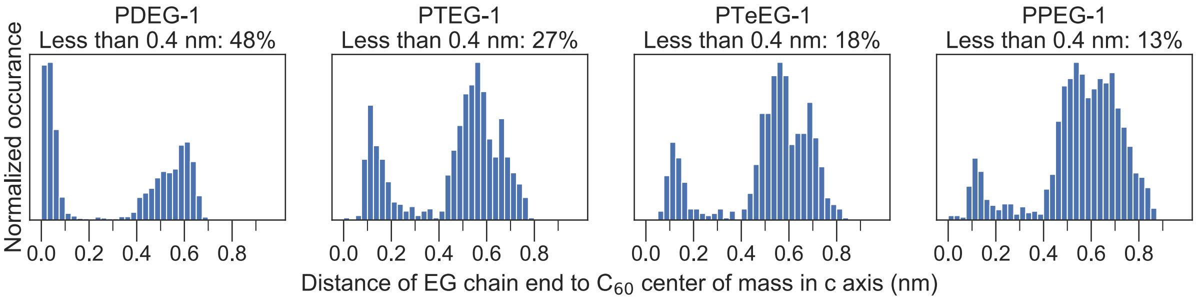

Fig. S4 Distribution of the distance between the end of the EG chain and the center of mass of the C_{60} along the *c* axis. The mean (μ) and the standard deviation (σ) are given for all molecules.

Table S3: The calculated average distance between the centre of mass of the fullerenes within the same bilayer.

	C60-C60 average distance (Å)
PDEG-1	9.88
PTEG-1	9.88
PTeEG-1	9.87
PPEG-1	9.89

NMR spectra

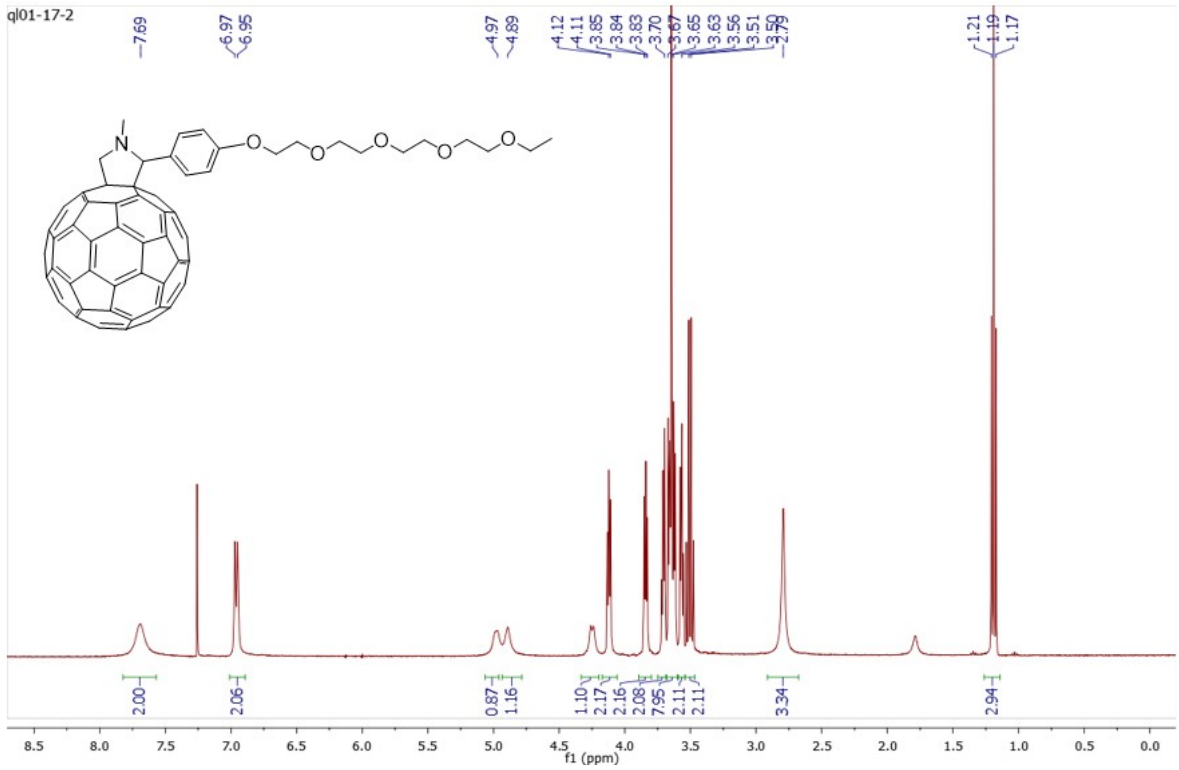

Fig. S5 ^{1}H NMR spectrum of PTeEG-1.

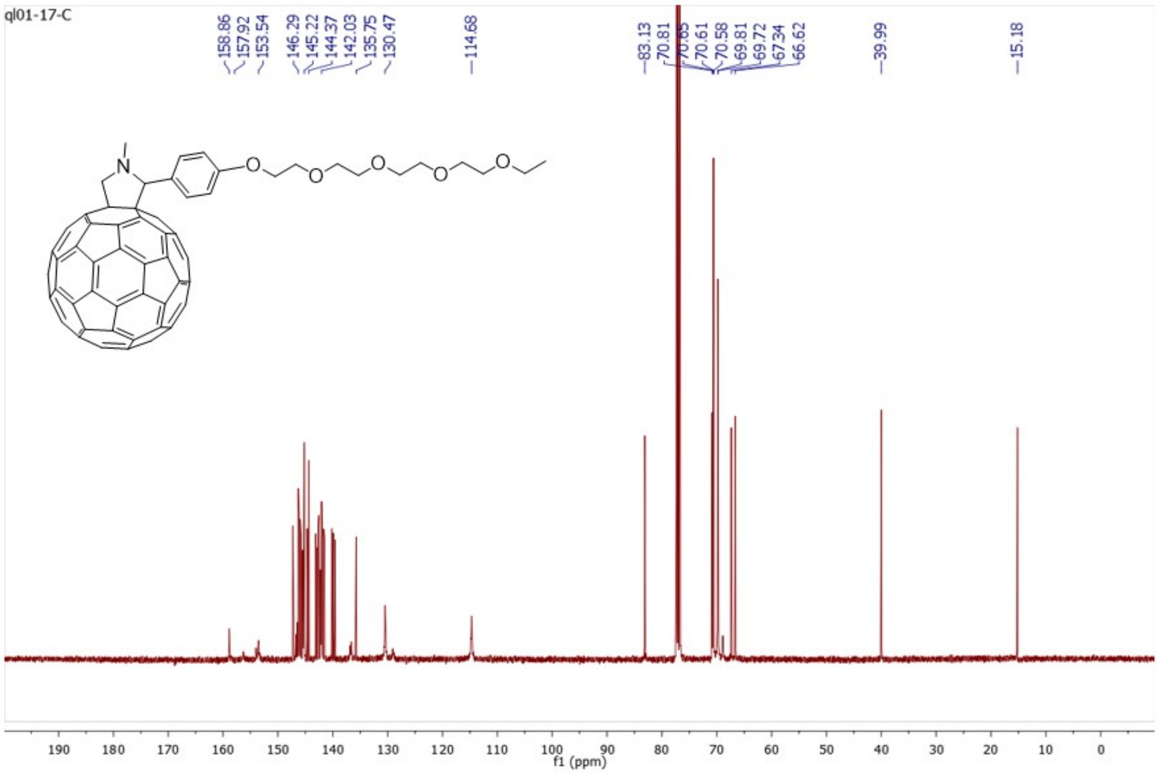

Fig. S6 ^{13}C NMR spectrum of PTeEG-1.

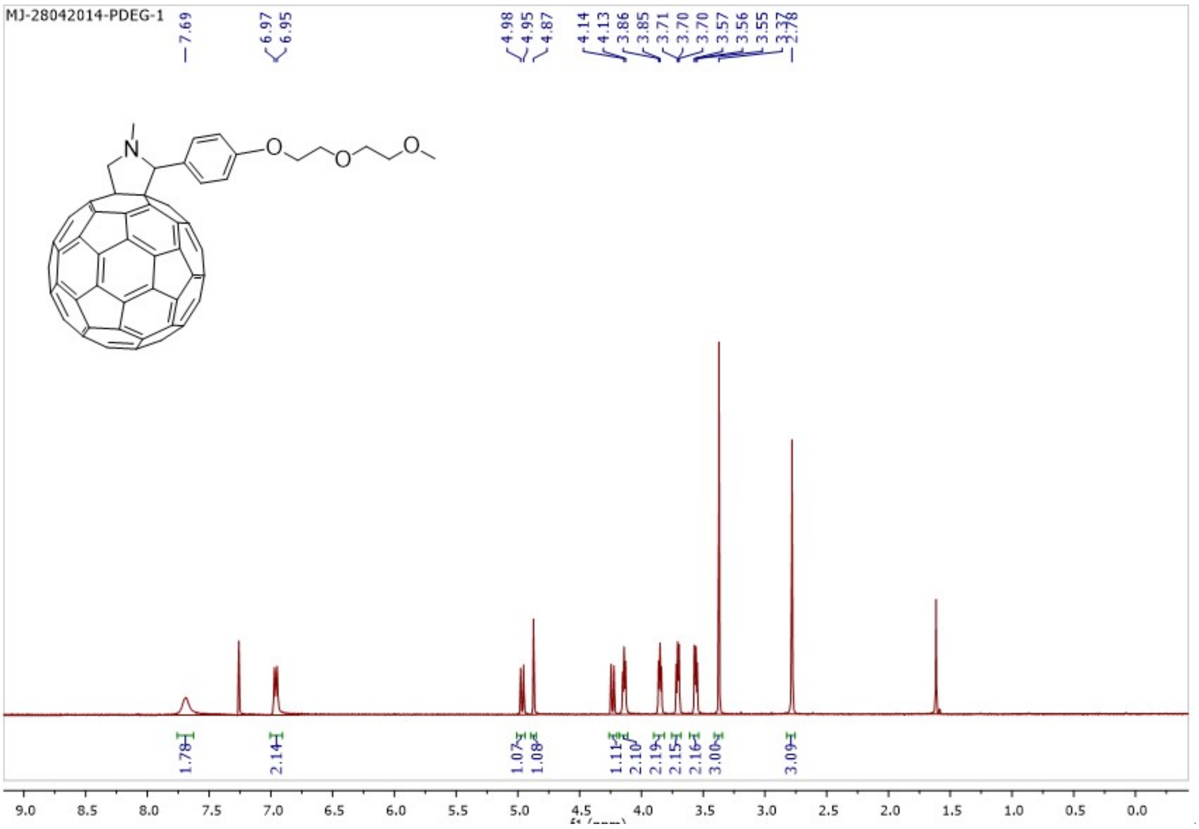

Fig. S7 ^{1}H NMR spectrum of PDEG-1.

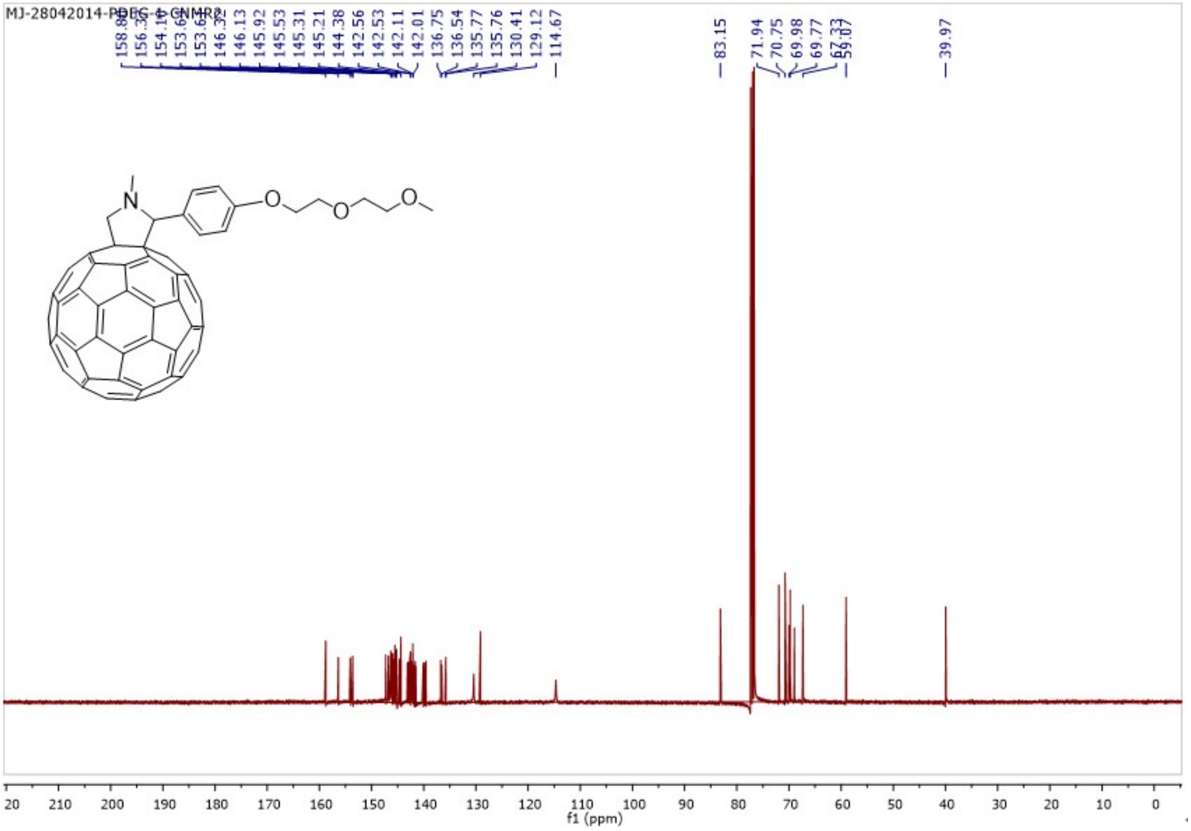

Fig. S8 ^{13}C NMR spectrum of PDEG-1.

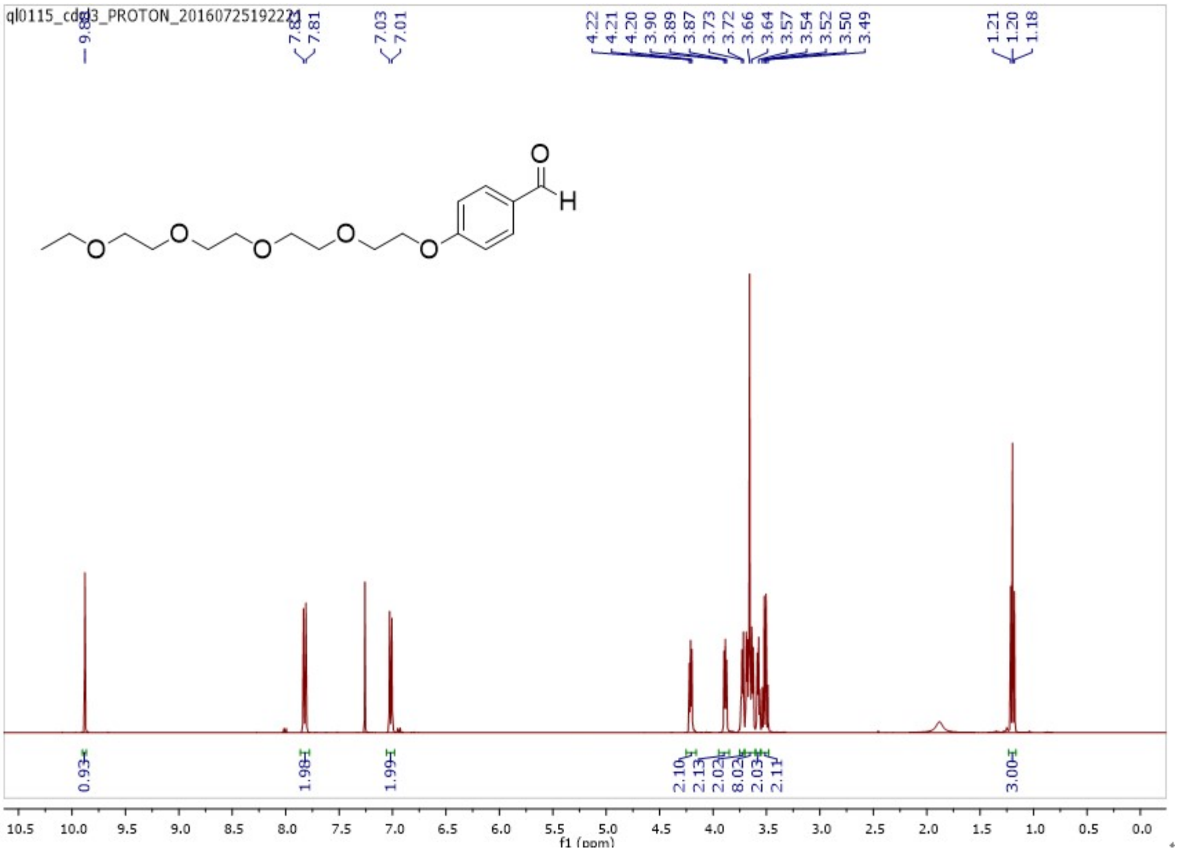

Fig. S9 ^{1}H NMR spectrum of ‘tegylated’ benzaldehyde **3**.

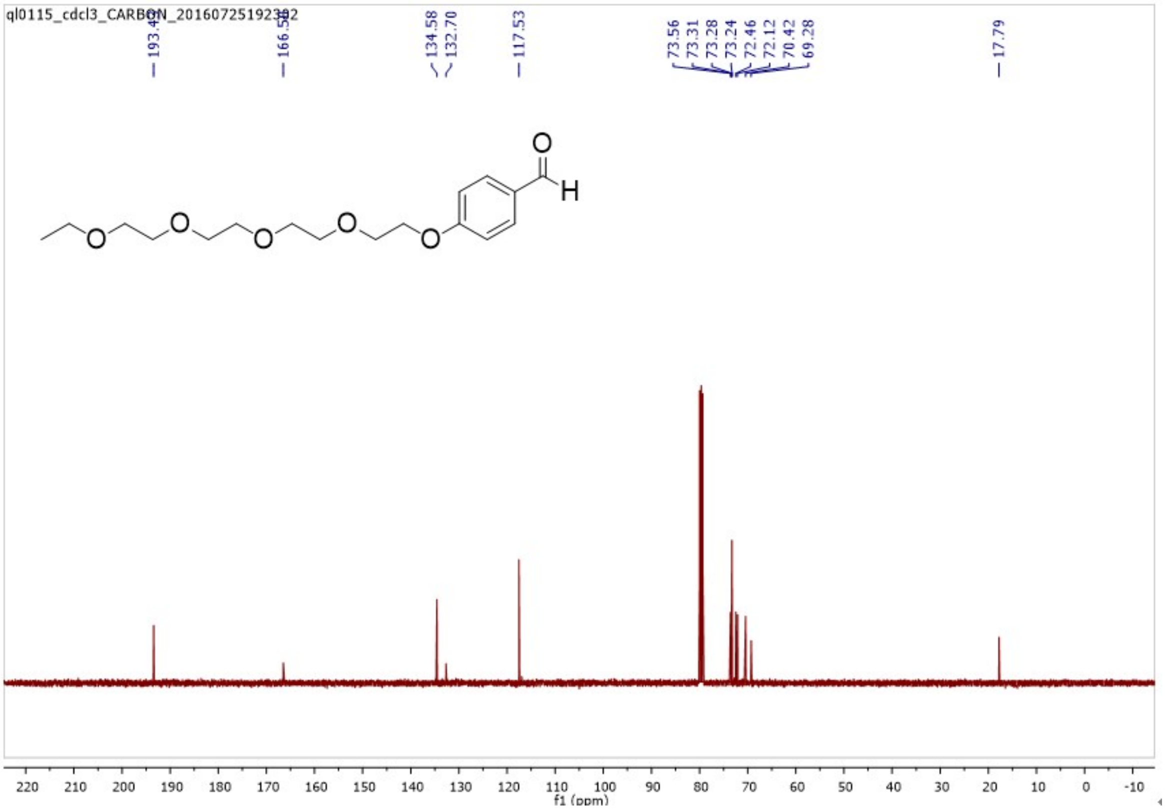

Fig. S10 ^{13}C NMR spectrum of ‘tegylated’ benzaldehyde **3**.

References

1 E. Dauzon, A. E. Mansour, M. R. Niazi, R. Munir, D. M. Smilgies, X. Sallenave, C. Plesse, F. Goubard and A. Amassian, *ACS Appl. Mater. Interfaces*, 2019, **11**, 17570–17582.

2 T. Darden, D. York and L. Pedersen, *J. Chem. Phys.*, 1993, **98**, 10089–10092.

3 H. J. C. Berendsen, J. P. M. Postma, W. F. Van Gunsteren, A. Dinola and J. R. Haak, *J. Chem. Phys.*, 1984, **81**, 3684–3690.

4 M. J. Abraham, T. Murtola, R. Schulz, S. Páll, J. C. Smith, B. Hess and E. Lindahl, *SoftwareX*, 2015, **1**–**2**, 19–25.
